# Encouraging prediction during production facilitates subsequent comprehension: Evidence from interleaved object naming in sentence context and sentence reading

**DOI:** 10.1080/17470218.2015.1131309

**Published:** 2016-06-01

**Authors:** Florian Hintz, Antje S. Meyer, Falk Huettig

**Affiliations:** 1 Centre for Language Studies, Radboud University, Nijmegen, The Netherlands; 2 Max Planck Institute for Psycholinguistics, Nijmegen, The Netherlands; 3 Donders Institute for Brain, Cognition, and Behaviour, Radboud University, The Netherlands

**Keywords:** Language production, Object naming, Self-paced reading, Prediction

## Abstract

Many studies have shown that a supportive context facilitates language comprehension. A currently influential view is that language production may support prediction in language comprehension. Experimental evidence for this, however, is relatively sparse. Here we explored whether encouraging prediction in a language production task encourages the use of predictive contexts in an interleaved comprehension task. In Experiment 1a, participants listened to the first part of a sentence and provided the final word by naming aloud a picture. The picture name was predictable or not predictable from the sentence context. Pictures were named faster when they could be predicted than when this was not the case. In Experiment 1b the same sentences, augmented by a final spill-over region, were presented in a self-paced reading task. No difference in reading times for predictive versus non-predictive sentences was found. In Experiment 2, reading and naming trials were intermixed. In the naming task, the advantage for predictable picture names was replicated. More importantly, now reading times for the spill-over region were considerable faster for predictive than for non-predictive sentences. We conjecture that these findings fit best with the notion that prediction in the service of language production encourages the use of predictive contexts in comprehension. Further research is required to identify the exact mechanisms by which production exerts its influence on comprehension.

A hallmark of human communication is the speed with which we process language. In dialogue, interlocutors typically react to previous turns very quickly, often within as little as 200 ms (cf. De Ruiter, Mitterer, & Enfield, 2006; Heldner & Edlund, 2010). It is likely that communication is so fast and efficient because language users rely heavily on supportive contexts (cf. van Berkum, Brown, Zwitserlood, Kooijman, & Haggort, 2005; Huettig, 2015; Kutas, DeLong, & Smith, 2011; Lupyan & Clark, 2015, for reviews).

A currently prominent view assumes that language production may support prediction in language comprehension (Dell & Chang, 2014; Pickering & Garrod, 2013). Evidence supporting this notion, however, is sparse (but see Gambi, Cop, & Pickering 2015; Mani & Huettig, 2012; Ito, Corley, Pickering, Martin, & Nieuwland, 2015, for a link between production-based prediction and reading). If production-based prediction plays an important role in comprehension, one would expect that contexts encouraging prediction in the service of language production should also facilitate language comprehension.

Indeed, previous research implies that production tasks can increase the use of predictive contexts during comprehension (compared to comprehension settings without a production task). Gollan et al. (2011; see also Griffin & Bock, 1998), for instance, observed faster naming latencies for objects depicting words that appeared in strongly predictable contexts than for those appearing in weakly predictable contexts. When the same sentences were used in an eye-tracked reading task, highly predictable targets were read faster than weakly predictable targets. Interestingly, however, based on a post hoc analysis, Gollan et al. reported that the facilitation effect was much larger in the naming task than in the reading task. In other words, when the task set involved production in addition to comprehending the first part of the sentence, the degree to which participants experienced facilitation was higher than when the task set involved only comprehension.

In the current study, we further explored the hypothesis that a task set encouraging prediction in a production task also encourages readers to use predictive contexts in a comprehension task, compared to a task set only involving comprehension. To that end, Dutch participants carried out two tasks, a cross-modal naming task and a self-paced reading task. The cross-modal naming task involved comprehending the first part of a spoken sentence and naming an object that was presented at the end of the recording to complete the sentence. The task thus comprised a production component in addition to comprehension. Self-paced reading only involved comprehension. The same sentences were used in both tasks and contained critical target nouns, which appeared in both predictable and non-predictable contexts. In contrast to Gollan et al. (2011), within the predictable condition we chose items that were not highly but moderately predictable.

Experiment 1 was run as a between-participants manipulation: In Experiment 1a, participants carried out the cross-modal naming task; in Experiment 1b, another sample of participants read the complete sentences including the target in a self-paced word-by-word fashion. We measured participants’ picture naming latencies and their reading times for the target words. To anticipate the main results, a substantial naming advantage was found on predictable over non-predictable trials in Experiment 1a. In Experiment 1b, we did not observe significant facilitation in the predictable condition (with our moderately predictable items) relative to the non-predictable condition. In Experiment 2, we interleaved naming and reading trials, appearing in random order. If a task set including prediction serving language production increases the likelihood of using predictive contexts in comprehension, we should observe facilitation on the reading trials of Experiment 2.

## Experiment 1

### Method

#### Participants

We estimated the required number of participants to be able to draw reliable statistical conclusions using G*Power (Faul, Erdfelder, Lang, & Buchner 2007) prior to running the experiment. Following the program's calculation (54 participants per experiment), which was based on the items’ mean cloze probability and range and the number of items per condition, 109 members of the subject panel of the Max Planck Institute for Psycholinguistics took part in Experiments 1a and 1b (Experiment 1a: 15 male, mean age = 21 years, *SD =* 3; an additional participant had to be excluded from the analysis because of extensive data loss; Experiment 1b: 10 males, mean age = 22 years, *SD =* 2). All were native speakers of Dutch and did not report any history of learning or reading disabilities or neurological or psychiatric disorders. The participants were paid for participating in the study. The ethics board of the faculty of social sciences of the Radboud University approved the study.

#### Stimuli

In both sub-experiments, the stimuli consisted of 40 target nouns, which appeared in simple predictable sentences (e.g., “De man breekt op dit moment een glas”, the man breaks at this moment a glass) and non-predictable sentences (e.g., “De man leent op dit moment een glas”, the man borrows at this moment a glass; see Appendix, for all items). All sentences were of the same structure: The subject position was filled by “the man”, and the adverbial “at this moment” separated verb and object. Using this “padding” between verb and target, we aimed to provide enough time for participants to generate predictions. In Dutch, the resulting sentence construction is deemed quite natural by native speakers.

Thirty-five additional native speakers of Dutch (mean age = 21 years, *SD =* 2) provided cloze probability ratings over the internet. Cloze probability was the proportion of participants who chose to complete a sentence fragment with the word in question. In the predictable items, the targets’ mean cloze probability was .39 (*SD =* .24; range: .06–.8); in the non-predictable items, it was zero.

Analyses were carried out on the length and frequency (using the SUBTLEX-NL database) of the verbs and objects. Raw frequencies were transformed to Zipf values, as suggested by van Heuven, Mandera, Keuleers, and Brysbaert (2014). Pairwise comparisons revealed that the predictable and the non-predictable verbs did not differ with regard to number of letters, *t*(39) = −1.467, *p* = .15. The analysis showed that the non-predictable verbs were more frequent than the predictable verbs (*t*(39) = −2.896, *p* = .006), which is most likely due to the non-predictable verbs’ less specific selectional restrictions. As we predicted facilitation effects for predictable rather than non-predictable items, this difference does not undermine our conclusions. The objects’ Zipf-transformed mean word frequency was 4.52 (*SD =* 0.54).

For Experiment 1a, the 80 sentences, including the target nouns, were spoken with neutral intonation at a normal pace by a female native speaker of Dutch. Recordings were made in a sound-damped booth, sampling at 44 kHz (mono, 16-bit sampling resolution) and stored on computer. The mean sentence duration was 2800 ms (*SD =* 214). A second version of each recorded sentence was created by cutting off the target noun. The mean duration of the cut sentences was 2076 ms (*SD* = 155). Depictions of the 40 target words were selected from the Snodgrass database (Snodgrass & Vanderwart, 1980) and were coloured in or drawn by an artist.

The same sentences were used in written form in Experiment 1b. Neutral prepositional phrases were added to each sentence (e.g., “De man breekt op dit moment een glas *van de collectie*”, the man breaks at this moment a glass from the collection) to be able to measure potential spill-over effects (Mitchell, 1994, for discussion). The two words following the target were the same in all sentences (“van de”, from the). In Experiment 1b, 30% of the sentences were followed by a comprehension question. Half of the questions focused on the verb of the just-read sentence; the other half focused on the object. Half of the questions required a yes-response.

#### Procedure

The 40 predictable and the 40 non-predictable items were evenly distributed across two lists, with none of the target nouns appearing twice on a list. Participants were randomly assigned one list and were seated in a sound-shielded room.

The spoken sentences in Experiment 1a were presented to the participants through loudspeakers. A trial was structured as follows: A central fixation dot appeared in the centre of the screen for 250 ms. The dot disappeared, and the playback of the sentence started. Coinciding with the end of the recording, participants saw a picture of the target word, which they were asked to name as quickly as possible. The picture remained on the screen for 2000 ms; the inter-trial interval was 1500 ms. Each participant was presented with all 40 trials on one list. The order of trials was pseudo-randomized prior to the experiment. All trials, including participants’ responses, were recorded in wav files for later analysis. Due to very low naming agreement for the pictures, we had to exclude the predictable and the non-predictable versions of four items. Naming latencies were calculated as the difference between the onset of the presentation of the critical object and the onset of participants’ responses.

The same experimental lists were used in Experiment 1b. A trial started with the presentation of the first word of the sentence, next to a number of underscores indicating the number of words to follow (i.e., “moving window” format). Upon pushing the space bar with their left hand, participants advanced to the next word, and the previous word was replaced with an underscore. They were instructed to read the sentences as fast as possible. On 30% of the trials a comprehension question was asked, which they answered by pushing the left (No) or right (Yes) button on the mouse using their right hand. On trials where no comprehension question was asked, participants advanced to the next trial by pushing a button on the mouse. Their responses to the comprehension questions showed that they read the sentences carefully (mean accuracy = 93%, *SD* = 8). Reading times for the target words and post-target words were calculated as the difference between the respective onsets of presentation and participants’ button presses.

### Results

Naming latencies and reading times (RTs) were log-transformed and analysed using linear mixed-effects regression models in R with simultaneous inclusion of participants and items as random factors. The full model included a fixed factor of condition (predictable vs. non-predictable) and random intercepts and slopes for condition by the random factors participants and items. This model was compared to the same model without the fixed effect of condition using a likelihood test. Including condition improved the model fit, χ^2^(2) = 23.583, *p* < .001, in the naming latency analysis in Experiment 1a. The full model revealed that participants named the target objects on average 96 ms faster when these were preceded by a predictable lead-in sentence than when they were preceded by a non-predictable lead-in sentence, β = −0.147, *SE* = 0.026, *t* = −5.64 (Table 1, for means). Applying the same analysis to the reading data revealed that the target words and the word following the target (spill-over region, henceforth) were read 7 ms and 8 ms faster, respectively, when preceded by a predictable verb than when preceded by a non-predictable verb. These differences were not statistically significant [target: β = −0.011, *SE* = 0.019, *t* = −0.55, χ^2^(2) = 0.311, *p* > .5; spill-over: β = −0.018, *SE* = 0.017, *t* = −1.01, χ^2^(2) = 1.035, *p* > .3].

**Table 1.** Mean naming latencies and (post-)target reading times for Experiments 1a, 1b, and 2

Dependent variable/Experiment	Experiment 1a	Experiment 1b	Experiment 2
*Naming latencies*			
Predictable	657 (193)	—	752 (215)
Non-predictable	753 (232)	—	839 (314)
*Reading times*			
Predictable	—	397 (159)	327 (177)
Non-predictable	—	404 (171)	332 (182)
*Reading times post-target word*			
Predictable	—	370 (108)	312 (135)
Non-predictable	—	378 (118)	331 (156)

*Note:* All times and latencies in milliseconds. Standard deviations in parentheses.

A correlation analysis between an item's cloze probability and its facilitation effect (predictable minus non-predictable naming latency/RT) revealed a significant positive relationship between both measures in Experiment 1a (*r* = .347, *n* = 36, *p* = .038) but only a trend towards significance in Experiment 1b (target: *r* = .214, *n* = 40, *p* = .186; spill-over: *r* = .129, *n* = 40, *p* = .427).

### Discussion

The results of Experiment 1a replicate previous findings (Gollan et al., 2011; Griffin & Bock, 1998) and show that naming latencies were substantially reduced for predictable relative to non-predictable items. This finding is in line with our hypothesis: When participants were asked to carry out a production task (object naming) in addition to comprehension, the likelihood of facilitatory processing was increased as compared to when they carried out a “pure” comprehension task. The lack of a significant prediction effect in Experiment 1b may appear surprising given previous successful applications of the self-paced reading paradigm to study anticipatory language processing (e.g., van Berkum et al., 2005, Experiment 3) but note that we chose moderately predictable items in our study to avoid potential ceiling effects.

In Experiment 2, we tested whether the likelihood of using a predictive context on reading trials could be increased by randomly interleaving naming and reading trials. This manipulation was motivated by two considerations: First, we wanted to rule out that the self-paced reading paradigm might not be sensitive to capture such effects. Second, if our assumption is correct, mixing naming and reading trials—that is, production and comprehension tasks—should increase participants’ likelihood of using predictive contexts when processing the target word on reading trials. For the naming task, we expected similar results to those in Experiment 1a.

## Experiment 2

### Method

#### Participants

Fifty-six native speakers of Dutch (11 male, mean age = 21 years, *SD =* 3) who had not participated in Experiment 1 or the norming study took part in Experiment 2. None of them reported any history of learning or reading disabilities or neurological or psychiatric disorders. Due to a programming error, two participants had to be excluded.

#### Stimuli and procedure

The materials were the same as those in Experiments 1a and 1b. The 80 naming items and the 80 reading items were evenly distributed across four lists, with each of the target nouns appearing only once on a list. Participants were randomly assigned lists. The order of trials was completely randomized in the beginning of a testing session. Apart from that, trial structure and procedure were identical to those in the previous experiments.

### Results and discussion

Naming data and reading data were analysed separately. As in Experiment 1b, accuracy in the comprehension questions on reading trials indicated that participants read the sentences carefully (mean accuracy = 91%, *SD* = 11).

The same statistical procedure as that in Experiment 1 was applied. The analysis revealed a naming advantage very similar to that observed in Experiment 1a: Participants were 87 ms faster at naming the target object when it was preceded by a predictable than when it was preceded by a non-predictable lead-in sentence (β = −0.106, *SE* = 0.018, *t* = −6.01), χ^2^(2) = 25.629, *p* < .001. However, on reading trials, we now observed a facilitatory effect as well: The spill-over region was read 19 ms faster on predictable than on non-predictable items. This difference was statistically significant (β = −0.047, *SE* = 0.017, *t* = −2.81), χ^2^(2) = 7.338, *p* = .007. No facilitation effect was found for target reading times (β = −0.015, *SE* = 0.019, *t* = −0.75), χ^2^(2) = 0.575, *p* > .4.

Correlation analyses, carried out as previously, showed a marginal significant positive relationship between an item's naming advantage and its cloze probability (*r* = .322, *n* = 36, *p* = .055). No significant correlation was found for reading trials (target: *r* = −.091, *n* = 40, *p* > .5; spill-over: *r* = .075, *n* = 40, *p* > .6).

## Conclusions

The present study supports the notion that prediction in the service of language production encourages the use of predictive contexts in comprehension: Substantial facilitation effects were observed when the participants’ task involved production (Experiments 1a and 2) but not when participants carried out a “pure” comprehension task (Experiment 1b).

One interpretation of the facilitation effects is that participants used their production system to anticipate predictable words not only on the production trials of both experiments, but also on the self-paced reading trials of Experiment 2. That is, they used their production system to predict the name of the object in the naming task and similarly used their production system to predict upcoming words in the self-paced reading task.

A possible objection to this interpretation of the results is that we observed the facilitation effect only in the spill-over region of Experiment 2, but not before the predictable word occurred. An alternative interpretation of our findings is therefore that this effect does not reflect a “downstream” consequence of production-based prediction, but merely facilitated integration of the target word with previous sentence context (cf. Van Petten & Luka, 2012). We cannot rule out such an account with certainty. Note that prediction effects in self-paced reading often manifest themselves in the spill-over region. Calvo and Castillo's (1996, Experiment 2) participants, for instance, read target words that confirmed or did not confirm the consequence of a preceding predictive context. The authors observed that the regions following the targets, but not the target words themselves, were read faster following predictive than following non-predictive contexts (see Mitchell, 1994, for a detailed discussion of this issue). Moreover, most authors of electrophysiological studies reporting reduced N400 effects on target words following predictable contexts also interpret such a result as reflecting prediction rather than facilitated integration of the target words (see Kutas et al., 2011, for further discussion).

One could argue that the sheer presence of a second task may have increased reading speed of the critical regions. Based on the current data, we cannot rule out this objection. However, such a general due task account is much less specific than our account and lacks a mechanism that would explain the facilitation effect. Why would the mere presence of a second task encourage prediction? A much more plausible interpretation is, in our opinion, that the increased likelihood of facilitated processing on reading trials is due to the specific nature of the production task. Specifically, we believe that a common prediction mechanism affected participants’ performance on both tasks. In other words, the increase of prediction in the service of language production may have increased the likelihood of facilitated processing on reading trials as well.

Why might encouraging prediction in a language production task facilitate integration in an interleaved comprehension task? We conjecture that processes involved in language production and dialogue contributed to the observed facilitation effects. In line with such a notion, recent electrophysiological evidence suggests that participants engaged in lexical processing when anticipating that an experimental confederate would produce an utterance (Baus et al., 2014). In a similar vein, using a joint naming paradigm involving two participants, Gambi et al. (2015) compared the coordination of two successive utterances within and between speakers. The authors observed that the way in which speakers produced their own utterances was affected by whether they anticipated the turn of their confederate. Thus, the coordination of speaker turns, a situation similar to the alternation between comprehension and production task sets in the present study, may make use of some mechanisms that are also involved in preparing to speak. Although our data do not unequivocally show that participants used their production system to anticipate upcoming words, we conjecture that it is the most parsimonious account of the data. Future research is required to confirm this interpretation.

To conclude, we have shown that the degree to which readers use predictive contexts is influenced by the task set: In our study, readers relied more on predictive contexts when they also carried out a production task that encouraged prediction but less so when they carried out a pure reading task.

## Appendix

### Target objects, predictable and non-predictable verbs. Cloze probability is provided for the predictable sentences

**Table:**

Target object	Predictable verb	Non-predictable verb	Cloze probability
appel (apple)	schillen (peel)	tekenen (draw)	.63
baard (beard)	scheren (trim)	zien (see)	.51
bal (ball)	trappen (kick)	lenen (borrow)	.6
band (tube)	verwisselen (change)	verliezen (loose)	.46
bank (coach)	bekleden (stiffen)	kiezen (choose)	.14
beker (cup)	winnen (win)	bekijken (look at)	.06
biertje (beer)	drinken (drink)	kopen (buy)	.6
bloem (flower)	planten (plant)	ontvangen (receive)	.06
boek (book)	publiceren (publish)	verstoppen (hide)	.14
boom (tree)	kappen (chop)	beschrijven (describe)	.69
boterham (sandwich)	smeren (prepare)	betalen (pay)	.77
broek (pants)	passen (fit)	zoeken (search)	.49
cadeau (present)	krijgen (receive)	stelen (steal)	.2
contract (contract)	ondertekenen (sign)	ontvangen (receive)	.69
deur (door)	openen (open)	zoeken (search)	.31
dief (thief)	arresteren (arrest)	filmen (film)	.34
doos (box)	tillen (lift)	verbergen (hide)	.23
fiets (bike)	repareren (repair)	pakken (grab)	.34
glas (glass)	breken (break)	lenen (borrow)	.2
hond (dog)	aaien (pet)	tekenen (draw)	.43
huis (house)	bezitten (own)	kiezen (choose)	.29
ijsje (ice-cream)	likken (lick)	overhandigen (hand over)	.77
kind (child)	beschermen (protect)	beschrijven (describe)	.43
lamp (lamp)	vervangen (replace)	verbergen (hide)	.31
muur (wall)	behangen (decorate)	bewaken (guard)	.8
overhemd (shirt)	strijken (iron)	zien (see)	.49
piano (piano)	stemmen (tune)	stelen (steal)	.23
pizza (pizza)	bestellen (order)	verkopen (sell)	.26
sigaar (cigar)	roken (smoke)	verstoppen (hide)	.29
sinaasappel (orange)	persen (squeeze)	overhandigen (hand over)	.71
standbeeld (statue)	onthullen (reveal)	bewaken (guard)	.17
stoel (chair)	verplaatsen (displace)	pakken (grab)	.14
taart (cake)	bakken (bake)	verkopen (sell)	.51
tafel (table)	dekken (prepare)	betalen (pay)	.66
tas (bag)	dragen (carry)	kopen (buy)	.09
touw (rope)	spannen (take up)	verliezen (lose)	.2
trein (train)	missen (miss)	filmen (film)	.06
varken (pig)	slachten (slaughter)	fotograferen (take a photo)	.46
vis (fish)	vangen (catch)	fotograferen (take a photo)	.23
wond (wound)	hechten (suture)	bekijken (look at)	.8
